# Supplementary material for: Impact of Antimicrobial-Resistant Bacterial Pneumonia on In-Hospital Mortality and Length of Hospital Stay: A Retrospective Cohort Study in Spain
Source: Antibiotics (Basel). 2025 Oct 10;14(10):1006. doi: 10.3390/antibiotics14101006 (PMC12561635; doi:10.3390/antibiotics14101006)
Supplement: Supplementary file 1 [file antibiotics-14-01006-s001.zip › Supplementary Materials File S1.pdf]

Supplementary Materials File S1. Balance Diagnostics Before and After 1:1 Propensity Score Matching (ATET in-hospital mortality)

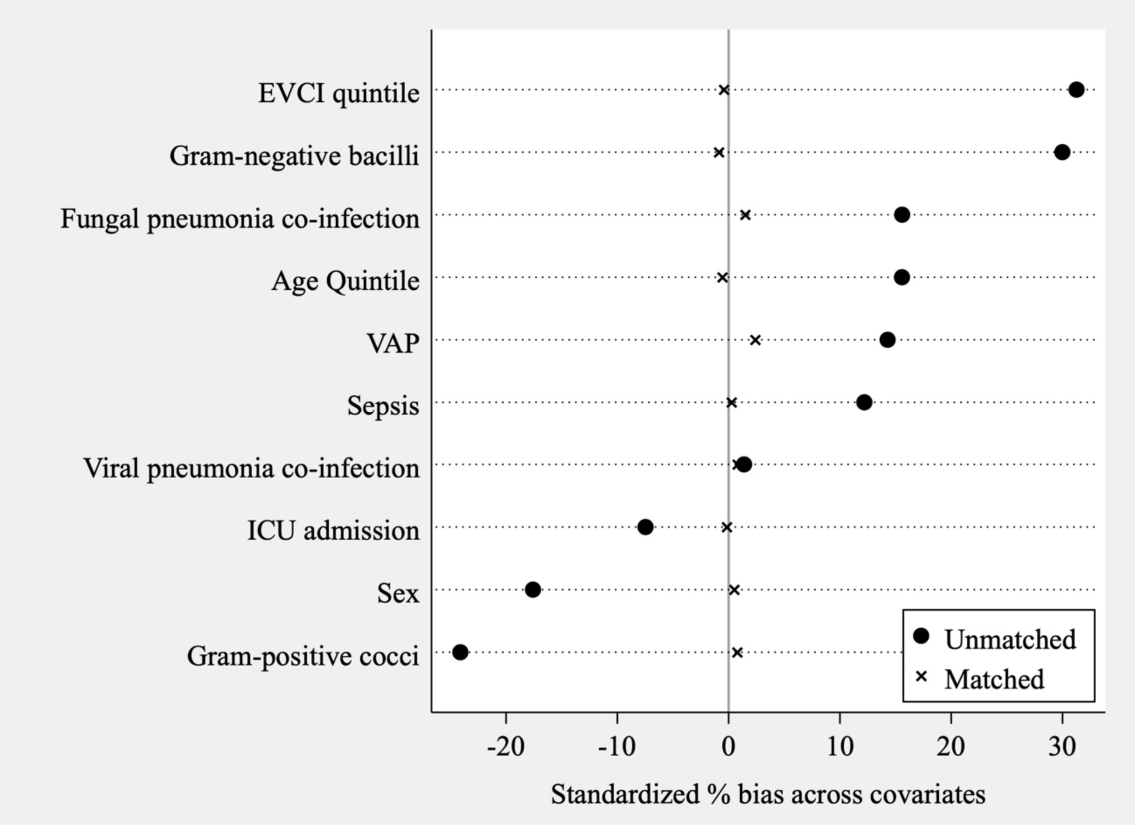

	Before Matching Std. Difference	After Matching Std. Difference	% Bias Reduction
Gran-negative bacilli	30.00%	0.90%	97.10%
Gram-positive cocci	24.10%	0.80%	96.80%
Age Quintile	15.60%	0.60%	96.30%
Sex	17.60%	0.50%	97.20%
EVCI	31.30%	0.40%	98.60%
Sepsis	12.20%	0.30%	97.90%
Fungal pneumonia co-infection	15.60%	1.50%	90.50%
Viral pneumonia co-infection	1.40%	0.80%	43.30%
VAP	14.30%	2.40%	83.30%
ICU admission	7.50%	0.20%	97.70%
Overall Mean Bias	16.90%	0.80%	95.30%

Matching Details: Method: 1:1 propensity score matching without replacement, Caliper: 0.1, Pairs: 6,017 case-control pairs, Matching Ratio: 1:1 exact, Estimate: Average Treatment Effect on the Treated (ATET) for in-hospital mortality.
EVCI, Elixhauser-van Walraven comorbidity index, VAP, ventilator-associated pneumonia; ICU, intensive care unit; Std., standard.
