# Supplementary material for: Impact of Antimicrobial-Resistant Bacterial Pneumonia on In-Hospital Mortality and Length of Hospital Stay: A Retrospective Cohort Study in Spain
Source: Antibiotics (Basel). 2025 Oct 10;14(10):1006. doi: 10.3390/antibiotics14101006 (PMC12561635; doi:10.3390/antibiotics14101006)
Supplement: Supplementary file 1 [file antibiotics-14-01006-s001.zip › Supplementary Materials File S2.pdf]

**Supplementary Materials File S2**. International Classification of Diseases, 10th Revision, Clinical Modification (ICD-10-CM-CM) codes for definition of bacterial pneumonia

| ICD-10-CM | Description | Observation |
|---|---|---|
| A01.03 | Pneumonia due to typhoidal *Salmonella* | Exclusion criteria (very unusual) |
| A02.22 | Pneumonia due to non- typhoidal *Salmonella* | Exclusion criteria (very unusual) |
| A15 | Respiratory tuberculosis (*Mycobacterium tuberculosis*) | Exclusion criteria (*Mycobacteriales* infection) |
| A20.2 | Pneumonic plague (*Yersinia pestis*) | Exclusion criteria (very unusual) |
| A21.2 | Pulmonary tularaemia (*Francisella tularensis*) | |
| A22.1 | Pulmonary anthrax (*Bacillus anthracis*) | |
| A23.0 | Brucellosis due to *Brucella melitensis* | |
| A23.1 | Brucellosis due to *Brucella abortus* | |
| A23.2 | Brucellosis due to *Brucella suis* | |
| A23.3 | Brucellosis due to *Brucella canis* | |
| A23.8 | Other brucellosis | |
| A23.9 | Brucellosis, unspecified | |
| A24 | Glanders and melioidosis (Infection due to *Pseudomonas mallei*, *Burkholderia mallei*, *Pseudomonas pseudomallei*, *Burkholderia pseudomallei*) | Exclusion criteria (very unusual) |
| A27 | Leptospirosis (*Leptospira interrogans*) | Exclusion criteria (*Spirochaetales* infection) |
| A31.0 | Pulmonary mycobacterial infection (*Mycobacterium avium, Mycobacterium intracellulare, Mycobacterium kansasii*) | Exclusion criteria (*Mycobacteriales* infection) |
| A37.01 | Whooping cough due to *Bordetella pertussis* with pneumonia | |
| A37.11 | Whooping cough due to *Bordetella parapertussis* with pneumonia | |
| A37.81 | Whooping cough due to other Bordetella species with pneumonia | |
| A37.91 | Whooping cough, unspecified species with pneumonia | |
| A42.0 | Pulmonary actinomycosis (*Actinomyces spp.*) | |
| A43.0 | Pulmonary nocardiosis (*Nocardia spp.*) | |
| A48.1 | Legionnaires' disease (*Legionella pneumophila*) | |
| A52.72 | Syphilis of lungs and bronchi (*Treponema pallidum*) | Exclusion criteria (*Spirochaetales* infection) |
| A54.84 | Gonococcal pneumonia (*Neisseria gonorrhoeae)* | |
| A69 .8 | Pneumonia due to spirochaetal | Exclusion criteria (*Spirochaetales* infection) |
| A78 | Q fever (Infection due to *Coxiella burnetii*) | |
| J13 | Pneumonia due to *Streptococcus pneumoniae* | |
| J14 | Pneumonia due to *Hemophilus influenzae* | |
| J15.0 | Pneumonia due to *Klebsiella pneumoniae* | |
| J15.1 | Pneumonia due to *Pseudomonas aureginosa* | |
| J15.20 | Pneumonia due to Staphylococcus, unspecified | |
| J15.211 | Pneumonia due to methicillin susceptible *Staphylococcus aureus* | |
| J15.212 | Pneumonia due to methicillin resistant *Staphylococcus aureus* | |
| J15.29 | Pneumonia due to other *Staphylococcus* | |
| J15.3 | Pneumonia due to *Streptococcus*, group B | |
| J15.4 | Pneumonia due to other Streptococci | |
| J15.5 | Pneumonia due to *Escherichia coli* | |
| J15.6 | Pneumonia due to other aerobic Gram-negative bacteria | |
| J15.7 | Pneumonia due to *Mycoplasma pneumoniae* | |
| J15.8 | Pneumonia due to other specified bacteria | Exclusion criteria (causative agent not specified/ identified) |
| J15.9 | Unspecified bacterial pneumonia | Exclusion criteria (causative agent not specified/ identified) |
| J16.0 | Chlamydial pneumonia (*Chlamydophila pneumoniae*) | |
