# Supplementary material for: Impact of Antimicrobial-Resistant Bacterial Pneumonia on In-Hospital Mortality and Length of Hospital Stay: A Retrospective Cohort Study in Spain
Source: Antibiotics (Basel). 2025 Oct 10;14(10):1006. doi: 10.3390/antibiotics14101006 (PMC12561635; doi:10.3390/antibiotics14101006)
Supplement: Supplementary file 1 [file antibiotics-14-01006-s001.zip › Supplementary Materials File S3.pdf]

Supplementary Materials File S3. International Classification of Diseases, 10th Revision, Clinical Modification (ICD-10-CM-CM) codes for definition other bacterial infectious disease.

ICD-10-CM Code	Type of Bacterial Infectious Disease	Description
A01.05	Acute infective osteomyelitis	Typhoid osteomyelitis
A02.24	Acute infective osteomyelitis	*Salmonella* osteomyelitis
A52.77	Acute infective osteomyelitis	Syphilis of bone and joint
A54.4	Acute infective osteomyelitis	Gonococcal infection of musculoskeletal system
H05.02	Acute infective osteomyelitis	Osteomyelitis of orbit
H70.0	Acute infective osteomyelitis	Acute mastoiditis
H70.21	Acute infective osteomyelitis	Acute petrositis
H70.8	Acute infective osteomyelitis	Other mastoiditis and related conditions
H70.9	Acute infective osteomyelitis	Unspecified mastoiditis
M46.2	Acute infective osteomyelitis	Osteomyelitis of vertebra
M46.3	Acute infective osteomyelitis	Infection of intervertebral disc (pyogenic)
M46.4	Acute infective osteomyelitis	Discitis, unspecified
M46.5	Acute infective osteomyelitis	Other infective spondylopathies
M86.0	Acute infective osteomyelitis	Acute hematogenous osteomyelitis
M86.1	Acute infective osteomyelitis	Other acute osteomyelitis
M86.2	Acute infective osteomyelitis	Subacute osteomyelitis
M86.8	Acute infective osteomyelitis	Other osteomyelitis
M86.9	Acute infective osteomyelitis	Osteomyelitis, unspecified
A01.04	Infective arthritis	Typhoid arthritis
A02.23	Infective arthritis	*Salmonella* arthritis
A39.83	Infective arthritis	Meningococcal arthritis
A54.42	Infective arthritis	Gonococcal arthritis
M00.0x	Infective arthritis	Staphylococcal arthritis and polyarthritis
M00.1x	Infective arthritis	Pneumococcal arthritis and polyarthritis
M00.2x	Infective arthritis	Other streptococcal arthritis and polyarthritis
M00.8x	Infective arthritis	Arthritis and and polyarthritis due to other bacteria
M00.9	Infective arthritis	Pyogenic arthritis, unspecified
M01.Xx	Infective arthritis	Direct infection of joint in infectious and parasitic diseases classified elsewhere
T84.5x	Infective arthritis	Infection and inflammatory reaction due to internal joint prosthesis
T84.6x	Infective arthritis	Infection and inflammatory reaction due to internal fixation device
T84.7x	Infective arthritis	Infection and inflammatory reaction due to other internal orthopedic prosthetic devices
A02.1	Sepsis-causing bacteria	Salmonella sepsis
A20.7	Sepsis-causing bacteria	Septicemic plague
A21.7	Sepsis-causing bacteria	Generalized tularemia
A22.7	Sepsis-causing bacteria	Anthrax sepsis
A26.7	Sepsis-causing bacteria	Erysipelothrix sepsis
A31.2	Sepsis-causing bacteria	Disseminated mycobacterium avium-intracellulare complex (DMAC)
A32.7	Sepsis-causing bacteria	Listerial sepsis
A39.2	Sepsis-causing bacteria	Acute meningococcemia
A39.4	Sepsis-causing bacteria	Meningococcemia, unspecified
A40.0	Sepsis-causing bacteria	Sepsis due to *Streptococcus,* group A
A40.1	Sepsis-causing bacteria	Sepsis due to *Streptococcus,* group B
A40.3	Sepsis-causing bacteria	Sepsis due to *Streptococcus pneumoniae*
A40.8	Sepsis-causing bacteria	Other streptococcal sepsis
A40.9	Sepsis-causing bacteria	Streptococcal sepsis, unspecified
A41.01	Sepsis-causing bacteria	Sepsis due to Methicillin susceptible *Staphylococcus aureus*
A41.02	Sepsis-causing bacteria	Sepsis due to Methicillin resistant *Staphylococcus aureus*
A41.1	Sepsis-causing bacteria	Sepsis due to other specified *Staphylococcus*
A41.2	Sepsis-causing bacteria	Sepsis due to unspecified *Staphylococcus*
A41.3	Sepsis-causing bacteria	Sepsis due to *Haemophilus influenzae*
A41.4	Sepsis-causing bacteria	Sepsis due to anaerobes
A41.50	Sepsis-causing bacteria	Gram-negative sepsis, unspecified
A41.51	Sepsis-causing bacteria	Sepsis due to *Escherichia coli*
A41.52	Sepsis-causing bacteria	Sepsis due to *Pseudomonas* spp.
A41.53	Sepsis-causing bacteria	Sepsis due to *Serratia* spp.
A41.59	Sepsis-causing bacteria	Other Gram-negative sepsis
A41.81	Sepsis-causing bacteria	Sepsis due to *Enterococcus* spp.

A41.89	Sepsis-causing bacteria	Other specified sepsis
A41.9	Sepsis-causing bacteria	Sepsis, unspecified organism
A42.7	Sepsis-causing bacteria	Actinomycotic sepsis
A54.86	Sepsis-causing bacteria	Gonococcal sepsis
I76	Sepsis-causing bacteria	Septic arterial embolism
R65.20	Sepsis-causing bacteria	Severe sepsis without septic shock
R65.21	Sepsis-causing bacteria	Severe sepsis with septic shock
R78.81	Sepsis-causing bacteria	Bacteremia
T80.211	Sepsis-causing bacteria	Bloodstream infection due to central venous catheter
T80.218	Sepsis-causing bacteria	Other infection due to central venous catheter
T80.219	Sepsis-causing bacteria	Unspecified infection due to central venous catheter
T81.12	Sepsis-causing bacteria	Postprocedural septic shock
T81.44	Sepsis-causing bacteria	Sepsis following a procedure
A01.01	Central nervous system infectious disease	Typhoid meningitis
A02.21	Central nervous system infectious disease	Salmonella meningitis
A17.0	Central nervous system infectious disease	Tuberculous meningitis
A20.3	Central nervous system infectious disease	Plague meningitis
A22.8	Central nervous system infectious disease	Anthrax meningitis
A27.81	Central nervous system infectious disease	Aseptic meningitis in leptospirosis
A32.11	Central nervous system infectious disease	Listerial meningitis
A32.12	Central nervous system infectious disease	Listerial meningoencephalitis
A39.0	Central nervous system infectious disease	Meningococcal meningitis
A39.81	Central nervous system infectious disease	Meningococcal encephalitis
A42.81	Central nervous system infectious disease	Actinomycotic meningitis
A42.82	Central nervous system infectious disease	Actinomycotic meningitis
A52.1	Central nervous system infectious disease	Symptomatic neurosyphilis
A54.81	Central nervous system infectious disease	Gonococcal meningitis
A54.82	Central nervous system infectious disease	Gonococcal brain abscess
A69.21	Central nervous system infectious disease	Meningitis due to Lyme disease
G00.0	Central nervous system infectious disease	Hemophilus meningitis
G00.1	Central nervous system infectious disease	Pneumococcal meningitis
G00.2	Central nervous system infectious disease	Streptococcal meningitis
G00.3	Central nervous system infectious disease	Staphylococcal meningitis
G00.8	Central nervous system infectious disease	Other bacterial meningitis
G00.9	Central nervous system infectious disease	Bacterial meningitis, unspecified
G01	Central nervous system infectious disease	Meningitis in bacterial diseases classified elsewhere
G04.2	Central nervous system infectious disease	Bacterial meningoencephalitis and meningomyelitis, not elsewhere classified
G05.3	Central nervous system infectious disease	Encephalitis and encephalomyelitis in diseases classified elsewhere
G05.4	Central nervous system infectious disease	Myelitis in diseases classified elsewhere
G06.0	Central nervous system infectious disease	Intracranial abscess and granuloma
G06.1	Central nervous system infectious disease	Intraspinal abscess and granuloma
G06.2	Central nervous system infectious disease	Extradural and subdural abscess, unspecified
A02.0	Intra-abdominal infectious disease	Salmonella enteritis
A03x	Intra-abdominal infectious disease	Shigellosis
A04.0	Intra-abdominal infectious disease	Enteropathogenic Escherichia coli infection
A04.1	Intra-abdominal infectious disease	Enterotoxigenic Escherichia coli infection
A04.2	Intra-abdominal infectious disease	Enteroinvasive Escherichia coli infection
A04.3	Intra-abdominal infectious disease	Enterohemorrhagic Escherichia coli infection
A04.4	Intra-abdominal infectious disease	Other intestinal Escherichia coli infections
A04.5	Intra-abdominal infectious disease	Campylobacter enteritis
A04.6	Intra-abdominal infectious disease	Enteritis due to Yersinia enterocolitica
A04.7x	Intra-abdominal infectious disease	Enterocolitis due to Clostridium difficile
A04.8	Intra-abdominal infectious disease	Other specified bacterial intestinal infections
A04.9	Intra-abdominal infectious disease	Bacterial intestinal infection, unspecified
A05x	Intra-abdominal infectious disease	Other Foodborne bacterial intoxication, not elsewhere classified
A09	Intra-abdominal infectious disease	Infectious gastroenteritis and colitis, unspecified
A22.2	Intra-abdominal infectious disease	Gastrointestinal anthrax
A36.89	Intra-abdominal infectious disease	Diphtheritic peritonitis
A52.74	Intra-abdominal infectious disease	Syphilis of liver and other viscera
A54.85	Intra-abdominal infectious disease	Gonococcal peritonitis
A74.81	Intra-abdominal infectious disease	Chlamydial peritonitis

D73.3	Intra-abdominal infectious disease	Abscess of spleen
K35.2	Intra-abdominal infectious disease	Acute appendicitis with generalized peritonitis
K35.3	Intra-abdominal infectious disease	Acute appendicitis with localized peritonitis
K35.80	Intra-abdominal infectious disease	Unspecified acute appendicitis
K35.89	Intra-abdominal infectious disease	Other acute appendicitis
K37	Intra-abdominal infectious disease	Unspecified appendicitis
K38.9	Intra-abdominal infectious disease	Disease of appendix, unspecified
K55.30	Intra-abdominal infectious disease	Necrotizing enterocolitis, unspecified
K55.31	Intra-abdominal infectious disease	Stage 1 necrotizing enterocolitis
K55.32	Intra-abdominal infectious disease	Stage 2 necrotizing enterocolitis
K55.33	Intra-abdominal infectious disease	Stage 3 necrotizing enterocolitis
K57.00	Intra-abdominal infectious disease	Diverticulitis of small intestine with perforation and abscess without bleeding
K57.01	Intra-abdominal infectious disease	Diverticulitis of small intestine with perforation and abscess with bleeding
K57.12	Intra-abdominal infectious disease	Diverticulitis of small intestine without perforation or abscess without bleeding
K57.13	Intra-abdominal infectious disease	Diverticulitis of small intestine without perforation or abscess with bleeding
K57.20	Intra-abdominal infectious disease	Diverticulitis of large intestine with perforation and abscess without bleeding
K57.21	Intra-abdominal infectious disease	Diverticulitis of large intestine with perforation and abscess with bleeding
K57.32	Intra-abdominal infectious disease	Diverticulitis of large intestine without perforation or abscess without bleeding
K57.33	Intra-abdominal infectious disease	Diverticulitis of large intestine without perforation or abscess with bleeding
K57.40	Intra-abdominal infectious disease	Diverticulitis of both small and large intestine with perforation and abscess without bleeding
K57.41	Intra-abdominal infectious disease	Diverticulitis of both small and large intestine with perforation and abscess with bleeding
K57.52	Intra-abdominal infectious disease	Diverticulitis of both small and large intestine without perforation or abscess without bleeding
K57.53	Intra-abdominal infectious disease	Diverticulitis of both small and large intestine without perforation or abscess with bleeding
K57.80	Intra-abdominal infectious disease	Diverticulitis of intestine, part unspecified, with perforation and abscess without bleeding
K57.81	Intra-abdominal infectious disease	Diverticulitis of intestine, part unspecified, with perforation and abscess with bleeding
K57.92	Intra-abdominal infectious disease	Diverticulitis of intestine, part unspecified, without perforation or abscess without bleeding
K57.93	Intra-abdominal infectious disease	Diverticulitis of intestine, part unspecified, without perforation or abscess with bleeding
K63.0	Intra-abdominal infectious disease	Abscess of intestine
K65.0	Intra-abdominal infectious disease	Generalized (acute) peritonitis
K65.1	Intra-abdominal infectious disease	Peritoneal abscess
K65.2	Intra-abdominal infectious disease	Spontaneous bacterial peritonitis
K65.3	Intra-abdominal infectious disease	Choleperitonitis
K65.8	Intra-abdominal infectious disease	Other peritonitis
K65.9	Intra-abdominal infectious disease	Peritonitis, unspecified
K68.1	Intra-abdominal infectious disease	Retroperitoneal abscess
K75.0	Intra-abdominal infectious disease	Abscess of liver
K80.00	Intra-abdominal infectious disease	Calculus of gallbladder with acute cholecystitis without obstruction
K80.01	Intra-abdominal infectious disease	Calculus of gallbladder with acute cholecystitis with obstruction
K80.12	Intra-abdominal infectious disease	Calculus of gallbladder with acute and chronic cholecystitis without obstruction
K80.13	Intra-abdominal infectious disease	Calculus of gallbladder with acute and chronic cholecystitis with obstruction
K80.18	Intra-abdominal infectious disease	Calculus of gallbladder with other cholecystitis without obstruction
K80.19	Intra-abdominal infectious disease	Calculus of gallbladder with other cholecystitis with obstruction
K80.30	Intra-abdominal infectious disease	Calculus of bile duct with cholangitis, unspecified, without obstruction
K80.31	Intra-abdominal infectious disease	Calculus of bile duct with cholangitis, unspecified, with obstruction
K80.32	Intra-abdominal infectious disease	Calculus of bile duct with acute cholangitis without obstruction
K80.33	Intra-abdominal infectious disease	Calculus of bile duct with acute cholangitis with obstruction
K80.36	Intra-abdominal infectious disease	Calculus of bile duct with acute and chronic cholangitis without obstruction
K80.37	Intra-abdominal infectious disease	Calculus of bile duct with acute and chronic cholangitis with obstruction
K80.40	Intra-abdominal infectious disease	Calculus of bile duct with cholecystitis, unspecified, without obstruction
K80.41	Intra-abdominal infectious disease	Calculus of bile duct with cholecystitis, unspecified, with obstruction
K80.42	Intra-abdominal infectious disease	Calculus of bile duct with acute cholecystitis without obstruction
K80.43	Intra-abdominal infectious disease	Calculus of bile duct with acute cholecystitis with obstruction
K80.46	Intra-abdominal infectious disease	Calculus of bile duct with acute and chronic cholecystitis without obstruction
K80.47	Intra-abdominal infectious disease	Calculus of bile duct with acute and chronic cholecystitis with obstruction
K80.60	Intra-abdominal infectious disease	Calculus of gallbladder and bile duct with cholecystitis, unspecified, without obstruction
K80.61	Intra-abdominal infectious disease	Calculus of gallbladder and bile duct with cholecystitis, unspecified, with obstruction
K80.62	Intra-abdominal infectious disease	Calculus of gallbladder and bile duct with acute cholecystitis without obstruction
K80.63	Intra-abdominal infectious disease	Calculus of gallbladder and bile duct with acute cholecystitis with obstruction
K80.66	Intra-abdominal infectious disease	Calculus of gallbladder and bile duct with acute and chronic cholecystitis without obstruction
K80.67	Intra-abdominal infectious disease	Calculus of gallbladder and bile duct with acute and chronic cholecystitis with obstruction
K80.80	Intra-abdominal infectious disease	Other cholelithiasis without obstruction

K80.81	Intra-abdominal infectious disease	Other cholelithiasis with obstruction
K81.2	Intra-abdominal infectious disease	Acute cholecystitis with chronic cholecystitis
K81.9	Intra-abdominal infectious disease	Cholecystitis, unspecified
K82.A1	Intra-abdominal infectious disease	Gangrene of gallbladder in cholecystitis
K82.A2	Intra-abdominal infectious disease	Perforation of gallbladder in cholecystit
K83.0x	Intra-abdominal infectious disease	Cholangitis
K83.2	Intra-abdominal infectious disease	Perforation of bile duct
K85.02	Intra-abdominal infectious disease	Idiopathic acute pancreatitis with infected necrosis
K85.12	Intra-abdominal infectious disease	Biliary acute pancreatitis with infected necrosis
K85.22	Intra-abdominal infectious disease	Pancreatitis aguda inducida por alcohol con necrosis infectada
K85.32	Intra-abdominal infectious disease	Drug induced acute pancreatitis with infected necrosis
K85.82	Intra-abdominal infectious disease	Other acute pancreatitis with infected necrosis
K85.92	Intra-abdominal infectious disease	Acute pancreatitis with infected necrosis, unspecified
A01.02	Infectious heart disease	Typhoid fever with heart involvement
A32.82	Infectious heart disease	Listerial endocarditis
A39.50	Infectious heart disease	Meningococcal carditis, unspecified
A39.51	Infectious heart disease	Meningococcal endocarditis
A39.52	Infectious heart disease	Meningococcal myocarditis
A39.53	Infectious heart disease	Meningococcal pericarditis
A52.03	Infectious heart disease	Syphilitic endocarditis
A52.06	Infectious heart disease	Other syphilitic heart involvement
A52.09	Infectious heart disease	Other cardiovascular syphilis
A54.83	Infectious heart disease	Gonococcal heart infection
I01.0	Infectious heart disease	Acute rheumatic pericarditis
I01.1	Infectious heart disease	Acute rheumatic endocarditis
I01.2	Infectious heart disease	Acute rheumatic myocarditis
I01.8	Infectious heart disease	Other acute rheumatic heart disease
I01.9	Infectious heart disease	Acute rheumatic heart disease, unspecified
I30.1	Infectious heart disease	Infective pericarditis
I33.0	Infectious heart disease	Acute and subacute infective endocarditis
I33.9	Infectious heart disease	Acute and subacute endocarditis, unspecified
I40.0	Infectious heart disease	Infective myocarditis
I40.9	Infectious heart disease	Acute myocarditis, unspecified
T82.6	Infectious heart disease	Infection and inflammatory reaction due to cardiac valve prosthesis
T82.7	Infectious heart disease	Infection and inflammatory reaction due to other cardiac and vascular devices, implants and grafts, initial encounter
A31.1	Skin and soft tissue infectious disease	Cutaneous mycobacterial infection
A36.3	Skin and soft tissue infectious disease	Cutaneous diphtheria
A43.1	Skin and soft tissue infectious disease	Cutaneous nocardiosis
A46	Skin and soft tissue infectious disease	Erysipelas
A48.0	Skin and soft tissue infectious disease	Gas gangrene
H00.03x	Skin and soft tissue infectious disease	Abscess of eyelid
H05.01x	Skin and soft tissue infectious disease	Cellulitis of orbit
H60.0x	Skin and soft tissue infectious disease	Abscess of external ear
H60.1x	Skin and soft tissue infectious disease	Cellulitis of external ear
I70.26x	Skin and soft tissue infectious disease	Atherosclerosis of native arteries of extremities with gangrene
I73.01	Skin and soft tissue infectious disease	Raynaud's syndrome with gangrene
I96	Skin and soft tissue infectious disease	Gangrene, not elsewhere classified
J34.0	Skin and soft tissue infectious disease	Abscess, furuncle and carbuncle of nose
J85.0	Skin and soft tissue infectious disease	Gangrene and necrosis of lung
K12.2	Skin and soft tissue infectious disease	Cellulitis and abscess of mouth
K61.0	Skin and soft tissue infectious disease	Anal abscess
K61.1	Skin and soft tissue infectious disease	Rectal abscess
K61.2	Skin and soft tissue infectious disease	Anorectal abscess
K61.3	Skin and soft tissue infectious disease	Ischiorectal abscess
K61.4	Skin and soft tissue infectious disease	Intrasphincteric abscess
K61.5	Skin and soft tissue infectious disease	Intrasphincteric abscess
L00	Skin and soft tissue infectious disease	Staphylococcal scalded skin syndrome
L01x	Skin and soft tissue infectious disease	Impetigo
L02x	Skin and soft tissue infectious disease	Cutaneous abscess, furuncle and carbuncle
L03x	Skin and soft tissue infectious disease	Cellulitis and acute lymphangitis
L04x	Skin and soft tissue infectious disease	Acute lymphadenitis

L05x	Skin and soft tissue infectious disease	Pilonidal cyst and sinus
L08.0	Skin and soft tissue infectious disease	Pyoderma
L08.1	Skin and soft tissue infectious disease	Erythrasma
L08.89	Skin and soft tissue infectious disease	Oth local infections of the skin and subcutaneous tissue
L08.9	Skin and soft tissue infectious disease	Local infection of the skin and subcutaneous tissue, unsp
L88	Skin and soft tissue infectious disease	Pyoderma gangrenosum
M72.6	Skin and soft tissue infectious disease	Necrotizing fasciitis
N48.21	Skin and soft tissue infectious disease	Abscess of corpus cavernosum and penis
N48.22	Skin and soft tissue infectious disease	Cellulitis of corpus cavernosum and penis
N61.1	Skin and soft tissue infectious disease	Abscess of the breast and nipple
N73.0	Skin and soft tissue infectious disease	Acute parametritis and pelvic cellulitis
N73.2	Skin and soft tissue infectious disease	Unspecified parametritis and pelvic cellulitis
A02.25	Urinary tract infectious disease	Salmonella pyelonephritis
A18.1	Urinary tract infectious disease	Tuberculosis of genitourinary system
A36.84	Urinary tract infectious disease	Diphtheritic tubulo-interstitial nephropathy
A36.85	Urinary tract infectious disease	Diphtheritic cystitis
A52.75	Urinary tract infectious disease	Syphilis of kidney and ureter
A52.76	Urinary tract infectious disease	Other genitourinary symptomatic late syphilis
A54.01	Urinary tract infectious disease	Gonococcal cystitis and urethritis, unspecified
A56.01	Urinary tract infectious disease	Chlamydial cystitis and urethritis
N10	Urinary tract infectious disease	Acute pyelonephritis
N13.6	Urinary tract infectious disease	Pyonephrosis
N15.1	Urinary tract infectious disease	Renal and perinephric abscess
N15.9	Urinary tract infectious disease	Renal tubulo-interstitial disease, unspecified
N30	Urinary tract infectious disease	Acute cystitis
N34	Urinary tract infectious disease	Urethritis and urethral syndrome
N39.0	Urinary tract infectious disease	Urinary tract infection, site not specified
N39.0	Urinary tract infectious disease	Urinary tract infection, site not specified
T83.5	Urinary tract infectious disease	Infection and inflammatory reaction due to prosthetic device, implant and graft in urinary system
M60.0	Infective myositis	Infective myositis
M65x	Infective synovitis/ tenosynovitis	Synovitis and tenosynovitis
M71.0x	Infective bursitis	Abscess of bursa
M71.1x	Infective bursitis	Other infective bursitis

“x” applies to all codes within the range.
