# Supplementary material for: Impact of Antimicrobial-Resistant Bacterial Pneumonia on In-Hospital Mortality and Length of Hospital Stay: A Retrospective Cohort Study in Spain
Source: Antibiotics (Basel). 2025 Oct 10;14(10):1006. doi: 10.3390/antibiotics14101006 (PMC12561635; doi:10.3390/antibiotics14101006)
Supplement: Supplementary file 1 [file antibiotics-14-01006-s001.zip › Supplementary Materials File S4.pdf]

Supplementary Materials File S4. Calculation of the Elixhauser-van Walraven comorbidity index according to Elixhauser comorbidities using the International Classification of Diseases, 10th Revision, Clinical Modification (ICD-10-CM) codes.

**Comorbidities Elixhauser**	**ICD-10-CM Coding Algorithms Comorbidity**	Elixhauser points	van Walraven points
Congestive heart failure	I09.9, I11.0, I13.0, I13.2, I25.5, I42.0, 142.5-I42.9, I43.x, I50.x, P29.0	1	7
Cardiac arrhythmias	I44.1-I44.3, I45.6, I45.9, I47.x-I49.x, R00.0, R00.1, R00.8, T82.1, Z45.0, Z95.0	1	2
Valvular disease	A52.0, I05.x-I08.x, I09.1, I09.8, I34.x-I39.x, Q23.0-Q23.3, Z95.2, Z95.4	1	7
Pulmonary circulation disorders	I26.x, I27.x, I28.0, I28.8, I28.9	1	3
Peripheral vascular disorders	I70.x, I71.x, I73.1, I73.8, I73.9, I77.1, I79.0, I79.2, K55.1, K55.8, K55.9, Z95.8, Z95.9	1	0
Hypertension, uncomplicated*	I10.x	1	12
Hypertension, complicated*	I11.x-I13.x, I15.x	1	5
Paralysis	G04.1, G11.4, G80.1, G80.2, G81.x, G82.x, G83.0-G83.4, G83.9	1	-1
Other neurological disorders	G10.x-G 13.x, G20.x-G22.x, G25.4, G25.5, G31.2, G31.8, G31.9, G32.x, G35.x-G37.x, G40.x, G41.x, G93.1, G93.4, R47.0, R56.x	1	4
Chronic pulmonary disease	I27.8, 127.9, J40.x-J47.x, J60.x-J67.x, J68.4, J70.1, J70.3	1	0
Diabetes, uncomplicated	E10.0, E10.1, E10.9, E11.0, E11.1, E11.9, E12.0, E12.1, E12.9, E13.0, E13.1, E13.9, E14.0, E14.1, E14.9	1	0
Diabetes, complicated	E10.2-E10.8, E11.2-E11.8, E12.2-E12.8, E13.2-E13.8, E14.2-E14.8	1	6
Hypothyroidism	E00.x-E03.x, E89.0	1	0
Renal failure	I12.0, I13.1, N18.x, NI9.x, N25.0, Z49.0-Z49.2, Z94.0, Z99.2	1	0
Liver disease	B18.x, I85.x, I86.4, I98.2, K70.x, K71.1, K71.3-K71.5, K71.7, K72.x-K74.x, K76.0, K76.2-K76.9. Z94.4	1	0
Peptic ulcer disease excluding bleeding	K25.7, K25.9, K26.7, K26.9, K27.7, K27.9, K28.7, K28.9	1	11
AIDS/H1V*	B20.x-B22.x, B24.x	1	5
Lymphoma	C81.x-C85.x, C88.x, C90.0, C90.2, C96.x	1	0
Metastatic cancer*	C77.x-C80.x	1	9
Solid tumour without metastasis*	C00.x-C26.x, C30.x-C34.x, C37.x-C41.x, C43.x, C45.x-C58.x, C60.x-C76.x, C97.x	1	4
Rheumatoid arthritis/ collagen vascular diseases	L94.0, L94.1, L94.3, M05.x, M06.x, M08.x, M12.0, M12.3, M30.x, M31.0-M31.3, M32.x-M35.x, M45.x, M46.1, M46.8, M46.9	1	0
Coagulopathy	D65-D68.x, D69.1, D69.3-D69.6	1	3
Obesity	E66.x	1	-4
Weight loss	E40.x-E46.x, R63.4, R64	1	6
Fluid and electrolyte disorders	E22.2, E86.x, E87.x	1	5
Blood loss anaemia	D50.0	1	-2
Deficiency anaemia	D50.8, D50.9, D51.x-D53.x	1	-2
Alcohol abuse	F10, E52, G62.1, I42.6, K29.2, K70.0, K70.3, K70.9, T51.x, Z50.2, Z71.4, Z72.1	1	0
Drug abuse	F11.x-F16.x, F18.x, F19.x, Z71.5. Z72.2	1	-7
Psychoses	F20.x, F22.x-F25.x, F28.x, F29.x, F30.2, F31.2, F31.5	1	0
Depression	F20.4, F31.3-F31.5, F32.x, F33.x, F34.1, F41.2, F43.2	1	-3

Elixhauser defined a comorbidity measure for use with administrative databases of hospitalized patients with 30 variables and van Walraven improved Elixhauser's prediction of mortality by assigning a specific weight to each of these comorbidities.
"x" applies to all codes within the range.
* A hierarchy was established between the following pairs of comorbidities, so that only the more severe comorbidity was counted. For example: If both uncomplicated diabetes and complicated diabetes are present, count only complicated diabetes.
