# Supplementary material for: Impact of Antimicrobial-Resistant Bacterial Pneumonia on In-Hospital Mortality and Length of Hospital Stay: A Retrospective Cohort Study in Spain
Source: Antibiotics (Basel). 2025 Oct 10;14(10):1006. doi: 10.3390/antibiotics14101006 (PMC12561635; doi:10.3390/antibiotics14101006)
Supplement: Supplementary file 1 [file antibiotics-14-01006-s001.zip › Supplementary Materials File S5.pdf]

Supplementary Materials File S5. International Classification of Diseases, 10th Revision, Clinical Modification (ICD-10-CM-CM) codes for risk factors definition.. International Classification of Diseases, 10th Revision, Clinical Modification (ICD-10-CM-CM) codes for risk factors definition.

ICD-10-CM Code	Risk factor	Description
Z16.1x	AMR- bacteria	Resistance to beta lactam antibiotics
Z16.2x	AMR-bacteria	Resistance to other antibiotics
5A1522	ECMO	Extra Corporeal Membrane Oxygenation
B37.1	Fungal pneumonia	Pulmonary candidiasis
B38.0	Fungal pneumonia	Acute pulmonary coccidioidomycosis
B38.1	Fungal pneumonia	Chronic pulmonary coccidioidomycosis
B38.2	Fungal pneumonia	Pulmonary coccidioidomycosis, unspecified
B38.7	Fungal pneumonia	Disseminated coccidioidomycosis
B38.9	Fungal pneumonia	Coccidioidomycosis, unspecified
B39x	Fungal pneumonia	Histoplasmosis
B44.0	Fungal pneumonia	Invasive pulmonary aspergillosis
B44.1	Fungal pneumonia	Other pulmonary aspergillosis
B44.7	Fungal pneumonia	Disseminated aspergillosis
B44.9	Fungal pneumonia	Aspergillosis, unspecified
B45.0	Fungal pneumonia	Pulmonary cryptococcosis
B45.7	Fungal pneumonia	Disseminated cryptococcosis
B46.0	Fungal pneumonia	Pulmonary mucormycosis
B59	Fungal pneumonia	Pneumocystosis
5A1935Z	Mecanic venthilation	Mecanic venthilation <24h consecutive
5A1945Z	Mecanic venthilation	Mecanic venthilation 24-96h consecutive
5A1955Z	Mecanic venthilation	Mecanic venthilation > 96h consecutive
Y95	Nosocomial	Nosocomial condition
J95.851	VAP	Ventilator associated pneumonia
B01.2	Viral pneumia	Varicella pneumonia
B05.2	Viral pneumia	Measles complicated by pneumonia
B06.81	Viral pneumia	Rubella pneumonia
B25.0	Viral pneumia	Cytomegaloviral pneumonitis
J12.0	Viral pneumia	Adenoviral pneumonia
J12.1	Viral pneumia	Respiratory syncytial virus pneumonia
J12.2	Viral pneumia	Parainfluenza virus pneumonia
J12.3	Viral pneumia	Human metapneumovirus pneumonia
J12.9	Viral pneumia	Viral pneumonia, unspecified
B97.4+	Viral pneumia	Respiratory syncytial virus as the cause of diseases classified elsewhere
J12.89	Viral pneumia	Other viral pneumonia
B34.2^	Viral pneumia (COVID-19)	Coronavirus infection, unspecified
B97.2^	Viral pneumia (COVID-19)	Coronavirus as the cause of diseases classified elsewhere
J12.81^	Viral pneumia (COVID-19)	Pneumonia due to SARS-associated coronavirus
J12.82	Viral pneumia (COVID-19)	Pneumonia due to coronavirus disease 2019
U07.1*	Viral pneumia (COVID-19)	COVID-19, virus identified.
U07.2*	Viral pneumia (COVID-19)	COVID-19, virus not identified
J09.X1	Viral pneumia (Flu)	Influenza due to identified novel influenza A virus with pneumonia
J10.0x	Viral pneumia (Flu)	Influenza due to other identified influenza virus with other respiratory manifestations
J11.0	Viral pneumia (Flu)	Influenza with pneumonia, virus not identified

“x” applies to all codes within the range.
* Addiction code J12.89 or J12.9 is required
+ Addiction code J12.89 or J12.9 is required
^ In SARS-Cov2 pandemic, until the creation of code U07.1 (2020) and J12.82 (2022) these codes were used to assign COVID-19 pneumonia condition
