# Supplementary material for: Impact of Antimicrobial-Resistant Bacterial Pneumonia on In-Hospital Mortality and Length of Hospital Stay: A Retrospective Cohort Study in Spain
Source: Antibiotics (Basel). 2025 Oct 10;14(10):1006. doi: 10.3390/antibiotics14101006 (PMC12561635; doi:10.3390/antibiotics14101006)

Supplementary Materials File S6. Marginal plots of predicted probability of in-hospital mortality, by strata (quintile) based on univariate logistic regression analysis.

In-hospital mortality vs age quintile

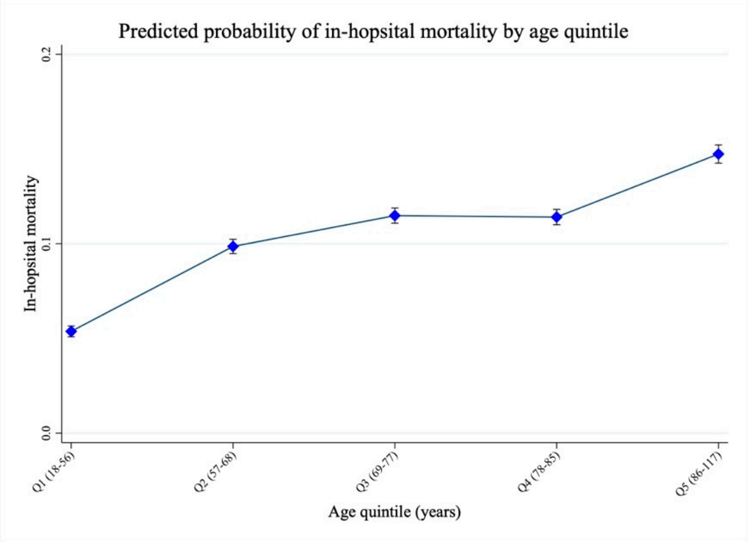

In-hospital mortality vs Elixhauser-van Walraven Comorbidity Index (EVCI) quintile

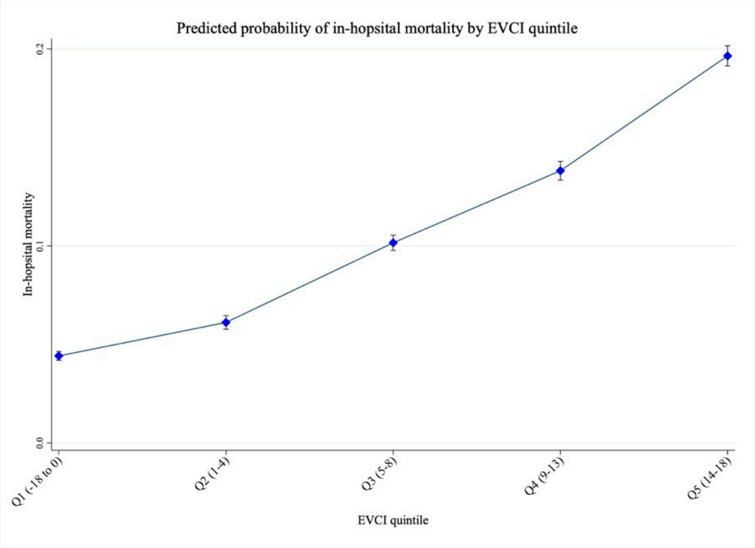

In-hospital mortality vs length of hospital stay (LOHS) quintile

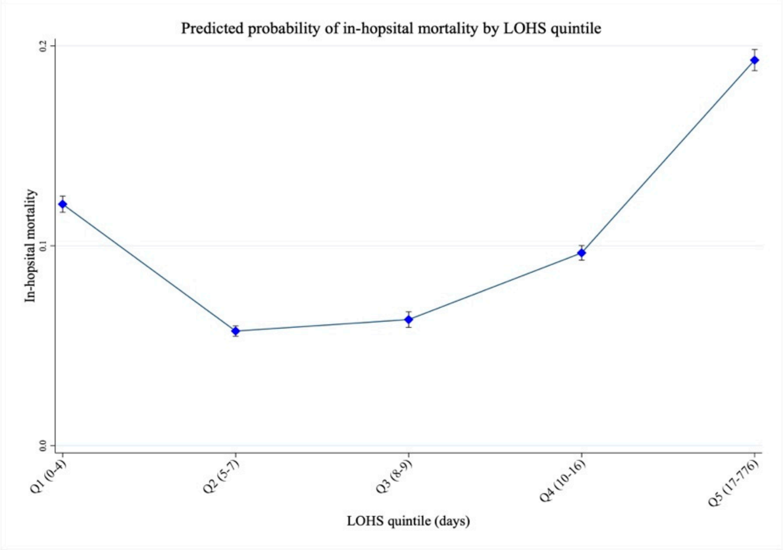

Supplement: Supplementary file 1 [file antibiotics-14-01006-s001.zip › Supplementary Materials File S6.pdf]
